# Progress of Surgery

**Published:** 1875-08

**Authors:** Jno. E. Owens

**Affiliations:** Lecturer on Surgery, Rush Medical College, Chicago


### Progress in Medical Sciences.

ARTICLE I.

### PROGRESS OF SURGERY.

BY JNO. E. OWENS, M.D.,

LECTURER ON SURGERY, RUSH MEDICAL COLLEGE, CHICAGO.

1. Treatment of Epistaxis. By Dr. BEVERLEY ROBINSON, of New York. (*Medical Record*, March 20, 1875 ; *The Monthly Abstract of Medical Sciences*, May, 1875.)

2. Division of the Isthmus to relieve Dyspnœa in Certain Cases of Bronchocele. By Sir G. DUNCAN GIBB, Bart., M.D., Physician to Westminster Hospital. (*Lancet*, Jan. 23, 1875 ; *The Monthly Abstract of Medical Sciences*, May, 1875.)

3. Removal of Goitre; Previous Ligature of Arteries. By Mr. LISTER. (*Brit. Med. Journal*, March 13, 1875 ; *The Med. News and Library*, May, 1875.)

4. Action of Salicylic Acid in Bacteria. (*Brit. Med. Journal*, March 6, 1875 ; *The Med. News and Library*, May, 1875.)

5. A Clinical Contribution to the Treatment of Tubal Pregnancy. By T. GAILLARD THOMAS, M.D. (*New York Med. Journal*, June, 1875.)

1. Dr. Robinson recommends, in obstinate epistaxis, compression of the facial arteries upon the superior maxillary bones, just before they reach the alæ of the nose, by means of two small pads made of lint. These are sewed to a piece of tape, the ends of which are passed across the cheeks and above the ears, and tied securely behind the occipital bone. Arrest of all cases of bleeding from the nose, by compression of the facial arteries is not claimed. The author trusts that this method may frequently be adjoined to other treatment with marked benefit to the patient, and by itself may prove of the greatest utility in exceptional circumstances where other means are not at hand. Dr. Robinson asserts that many of the worst cases of epistaxis originate in the septum, which receives its arteries mainly from the terminal branches of the facial.

2. The method suggested by Dr. Gibb has for its object the relief of dyspnœa from pressure upon the trachea in cases of enlargement of the thyroid gland affecting one or both lateral lobes, and implicating the isthmus. When the latter is enlarged, dyspnœa usually becomes a feature of great significance and the precursor of future mischief. If not relieved by treatment, the enlarged lateral lobes may extend on either side of the trachea itself and completely encircle it. In consequence of this, the tube is compressed laterally, and its form becomes oval, with a very narrow passage to breathe through. Such cases sooner or later end fatally. In the first case cited, the autopsy revealed the fact that a narrow oval fissure was all that the patient could breathe through during life. However, in some of our hospital museums, says Dr. Gibb, the preparations themselves show how hopeless are all means of cure when once the trachea is grasped by the tumor. Having long thought over the matter, he came to the conclusion that the only remedy in such cases is to remove or divide that portion of the bronchocele which was in contact with the trachea itself —the isthmus—*before* it had commenced either to encircle the tube or had become too firmly adherent to it. The second case reported illustrates the truly wonderful relief afforded by the operation.

In this case, under the influence of chloroform, an incision was made through the integuments in the median line over the trachea, about two inches long, the superficial fascia and then the deep were divided on a director, and finally, the surface of the tumor was exposed, which in size approached that of a flattened walnut. The isthmus on either side of this was more slender and smaller in bulk than the tumor, which, by means of an aneurism needle, permitted the application of a strong ligature on either side of it. The isthmus was then freed to the inner side of each ligature, divided, and wholly removed, the trachea being observed to be quite free.

There was not much bleeding, although several small veins ramified on the surface of the tumor. Subsequent examination showed the removed isthmus to be partly cystic in its character. The results in other cases fully justified the hopeful view entertained of the operation.

3. On the 6th of March, Mr. Lister removed a goitre from a young woman, having previously ligated the four principal arteries of supply to the thyroid, through a central incision, before the operation of removal was attempted. This has been found most effectual in restraining the hæmorrhage, which, without this precaution, is so serious a drawback to the operation. The patient is doing well.

4. Dr. Ludwig Letzeritz has lately tested the effect of salicylic acid on bacteria and micrococci, by placing under the microscope portions of fluid containing these organisms in abundance, and allowing solutions of salicylic acid to come into contact with them. He used solutions of four degrees of strength, viz., one part of salicylic acid and one of spirit, in 120, 90, 60, and 40 parts of water. The first two arrested the movements of the bacteria gradually; while, with the two stronger solutions, the arrest of movement was instantaneous. Although he has used it locally and internally with some benefit in diphtheria, he observes that more extensive observations are necessary in order to determine its value in that disease.

5. I can only refer to that part of Dr. Thomas' interesting paper which describes the brilliant operation but recently performed. Before detailing the treatment of the case, he remarks that few cases of extra-uterine pregnancy have, during their early and progressive stages, been brought to a favorable conclusion by surgical means. For this, he sets forth four good reasons: 1. The doubt which usually attends diagnosis; 2. The danger of attending invasion of the peritoneum; 3. The dangers arising from septic absorption from retention of the fœtus or its envelopes; 4. The certainty of grave hæmorrhage from opening into the extraordinarily vascular nest in which the fœtus is contained. To meet the indications, one of the following plans has been generally adopted: 1. Gastrotomy has been practiced, that the fœtal mass might be removed like an ovarian tumor; 2. The liquor amnii has been drawn off by a very delicate trocar, in order to diminish tension and check the growth of the cyst; 3. The fœtus has been killed in situ by the passage of strong currents of electricity, or the injection into the sac of strong narcotics, like atropia or morphia, with the hope that nature might eventually cause its discharge, with the contents of the abscess which it usually creates.

The first procedure is attended by the dangers of peritonitis and hæmorrhage; and the second and third by those of hæmorrhage into the sac, septicæmia, and subsequent formation and discharges of an abscess located just under the peritoneum. By the process proposed and adopted by Dr. Thomas, he hoped to avoid the dangers of peritonitis by opening the fœtal sac by the vagina, passing up to it between the folds of the broad ligaments. He seeks to prevent hæmorrhage by cutting into the sac by means of a knife rendered incandescent by a powerful current of electricity. By the complete removal of both fœtus and placenta; and by drainage of the sac, and by the use of carbolized injections through a tube of glass or silver, kept in it, and discharging its contents by the vagina, he avoids septicæmia. Dr. Thomas has put the plan to the test in the following manner:

The patient having been ætherized, was placed upon a table before a window admitting a strong light, in the left lateral position, and Sims' speculum introduced. Through this the cyst to the left of the uterus could be distinctly palpated. Now, fixing a long-handled tenaculum in the cervix uteri, and another in the vagina, near the left ilium, this part was by them put on a stretch so as to make of that side of the canal a triangle, the base of which was over the cyst, and the apex at the vulva. Taking the platinum knife of the galvano-caustic battery, which was brought to a white heat, the operator now passed it gently over the base of the triangle described as created in the vagina, carrying it from one tenaculum to the other. By repeating this the vaginal wall over the lower segment of the cyst was slowly cut through. In six minutes the cyst was opened by the incandescent knife, and a straw-colored, slightly pinkish fluid was thrown out with considerable force. Thus far no blood whatever had been lost. With the index finger in the cyst, the fœtus was felt lying horizontally, with the head towards the ilium and the feet towards the uterus. The steps of delivering the child exactly resembled those adopted in ordinary podalic version. The final delivery of the head was facilitated by the employment of a pair of long-handled placental forceps. The cord was then cut, and an attempt made to deliver the placenta by gentle traction, and detachment, as is done after ordinary labor. At this point, the first difficulty showed itself. When a little over half of the placenta had been separated, a very severe hæmorrhage took place, and so much was the patient's condition depreciated by it in the two or three minutes of its duration, that the operator was unwilling to delay for the removal of more. Tearing the detached portion off, a large gum-elastic catheter was passed into the sac and the latter injected with a solution of the persulphate of iron. The flow of blood was instantly checked, but the sac was left full of coagulated blood, and the remains of the placenta. A long tent of carbolized cotton, saturated with a solution of persulphate of iron, was substituted for the drainage tube. All went well till the evening of the fourth day, when symptoms of septicæmia showed themselves. These yielded to constantly repeated injections of carbolized water, at the end of a week. On the fifteenth day the remaining portion of the placenta came away spontaneously. On the sixteenth day evidences of an embolus in an unimportant vessel of the arm showed themselves, which created a small abscess. Ten weeks after the operation, Dr. Thomas had the satisfaction of seeing his patient in her parlor receiving company.

